# EGEFACE: A new face memory test with static and dynamic images

**DOI:** 10.3758/s13428-024-02592-0

**Published:** 2025-01-21

**Authors:** Sonia Amado, Murat C. Karataş, Elif Yüvrük, Aycan Kapucu

**Affiliations:** 1 Department of Psychology, Ege University, İzmir, Türkiye; 2 Department of Psychology, Bursa Technical University, Yıldırım, 16320 Bursa, Türkiye; 3 Department of Psychology, Mugla Sitki Kocman University, Muğla, Türkiye

**Keywords:** Face memory test, Unfamiliar face recognition, Individual differences, Ege face memory test

## Abstract

Face memory is a crucial cognitive ability necessary for maintaining a healthy social life. Recent studies reveal large individual differences in face recognition ability. Face memory tests are used to evaluate this ability. The main purpose of this study was to develop a new face memory test (EGEFACE) addressing the limitations of existing tests using both static and dynamic stimuli to increase ecological validity; employing face recognition algorithms to adjust test difficulty; measuring face memory accuracy independently of response bias by including both target-absent and target-present trials and using ROC analysis; and developing a test to measure both ends of the face recognition ability spectrum. After building a new database of static and dynamic faces, we created three difficulty levels using a face recognition algorithm. We collected data from 703 participants in two steps and examined the internal consistency, split-half reliability, and item–total score correlations. The reliability analysis confirmed that both target-absent and target-present trials of EGEFACE were reliable. High EGEFACE performers scored near super recognizer levels on CFMT+, while low performers showed limited overlap with prosopagnosic-level performance on CFMT+, suggesting EGEFACE’s sensitivity across different levels of face recognition ability. Overall, results indicated a moderate positive correlation between EGEFACE and CFMT+, showing that both tests assess similar cognitive skills, while a low to moderate correlation with KFMT suggests that EGEFACE measures cognitive ability that is related to yet distinct from face perception. The results suggest that EGEFACE shows promise as an ecologically valid and effective alternative tool for assessing individual differences in face memory.

Face recognition is a fundamental cognitive ability needed to sustain everyday life. Recent studies have focused on the individual differences in this ability (DeGutis et al., 2023; Megreya & Bindemann, 2013), as it varies considerably in the population. Face recognition ability is assumed to be normally distributed in the population, varying from developmental prosopagnosics, who suffer from selective impairment in face recognition without accompanying neurological distortion (Bowles et al., 2009), to super face recognizers, the people with extraordinary face recognition abilities (Russell et al., 2009). The development of new measurements that cover the entire range of face recognition ability has been an important methodological concern in the field. Additionally, identifying both ends of the spectrum has practical importance such that individuals with prosopagnosia may require support, while those with exceptional face recognition abilities could potentially pursue career opportunities in security-related fields (Bobak et al., 2016; Phillips et al., 2018; Ramon et al., 2019).

Face memory and face matching tests are used to evaluate individual differences in face recognition ability. Indeed, face memory and face matching are related but different abilities (Wilhelm et al., 2010). Face matching involves face perception but not memory, while face memory involves both face perception and memory (Fysh, 2018). In *face matching* tests, face stimuli are presented simultaneously, and participants are asked whether the identities of the faces match. For instance, the Kent Face Matching Test (KFMT; Fysh & Bindemann, 2018) and the Glasgow Face Matching Test (Burton et al., 2010) are widely used face matching tests using this general design. Therefore, face matching does not include a memory component. On the other hand, in the *face memory* tests, participants’ memory ability is tested for face stimuli that they have studied in a previous study phase. The Benton Facial Recognition Test (Benton, 1990) and Cambridge Face Memory Test (CFMT; Duchaine & Nakayama, 2006) are widely used face memory tests. Since these face memory tests were primarily designed to identify the lower end of the spectrum, namely those with developmental prosopagnosia, they may not be comprehensive enough to identify individuals with exceptional face recognition abilities (Russell et al., 2009). Therefore, face memory tests that were capable of identifying the high end of the spectrum were developed. For instance, the Cambridge Face Memory Test–Long Form (CFMT+; Russell et al., 2009), University of New South Wales Face Test (UNSW Face Test; Dunn et al., 2020), and Goldsmiths Unfamiliar Face Memory Test (GUFMT; Jansari et al., 2020) were developed to measure the entire range of the face recognition ability including super recognizers. However, as discussed below in detail, those widely used face memory tests have some important drawbacks (Esins et al., 2016). To address these concerns, we introduce the Ege Face Memory Test (EGEFACE) as an alternative test for assessing face memory performance in a more ecologically valid way.

In the following sections, we will begin by discussing the shortcomings of widely used face memory tests, followed by specific examples of tests that exhibit these issues, and then explore how EGEFACE was designed to address and overcome these deficiencies. To provide a preview, three common challenges are associated with face memory tests. One key issue is the lack of dynamic face stimuli. Most tests rely on static images, which fail to capture the natural variations in a person’s appearance. In contrast, dynamic stimuli that incorporate facial movements allow for a richer mental representation and enhance memory recognition. Another challenge involves the methods used to increase test difficulty. While techniques like adding noise or emotional expressions can make tests more challenging, they also risk introducing response biases that may confound the accuracy of performance. As an alternative, the use of face recognition algorithms has been proposed to adjust difficulty in a more reliable manner. Finally, the psychometric limitations of these tests are discussed in detail, addressing key aspects such as test–retest reliability and internal consistency.

The EGEFACE test aims to address these problems by (i) including dynamic stimuli and employing algorithms to adjust the difficulty, (ii) using receiver operating characteristic (ROC) analysis to measure test performance independently of potential response bias effects, and (iii) including target-absent trials that are critical for distinguishing between super recognizers and controls.

## Cambridge face memory test

CFMT is the most commonly used test to assess prosopagnosia (Burns et al., 2022). The test consists of three blocks, with a total of 72 trials. Six target identities are repeatedly presented in the test. Therefore, the identical target face stimulus is presented repeatedly, approximately 10 times throughout the test. Forty-six distractor face stimuli are also used repeatedly in the CFMT. Yet, developing an internal representation through repeated exposure to target and distractor faces may result in a familiarity disadvantage in memory, thereby increasing participants’ tendency to make “old” responses—namely, liberal response bias—regardless of actual item status, due to enhanced processing fluency for both types of items (e.g., Gold et al., 2007; Kramer & Berry, 2020; Kurilla, 2011). Thus, using each face stimuli only once in the test phase may methodologically diminish this potential response bias confound (see pages 9 and 13 for further discussion on response bias).

Face stimuli are presented without any contextual cues in CFMT. Despite CFMT’s choice of using highly standardized face images trimmed to remove hair and ears in order to increase experimental control, contextual information is known to affect face recognition performance (Megreya & Bindemann, 2018). Moreover, different facial features contribute to face recognition ability with different levels of importance (Towler et al., 2017). For example, when examining the effectiveness of a feature-by-feature comparison strategy in face matching tasks, Towler et al. (2017) found that ears were reported to be the most identifying feature, which were fully cropped in CFMT.

Only male face images are presented in CFMT to increase experimental control. However, studies show that face memory is affected by own-group bias, indicating superior face memory performance towards members of the group participants also belong to (Rollins et al., 2020; Strickland-Hughes et al., 2020; Young et al., 2010). Using only male images in CFMT might create a disadvantage for female participants. Although the Cambridge Face Memory Test–Female Long Form (F-CFMT+; Arrington et al., 2022) has been developed recently, this test still suffers from the same problem, as it only includes female images. Therefore, both male and female face stimuli must be included to control for potential confound of own-group bias.

Moreover, face stimuli are presented in grayscale in CFMT to increase experimental control, compromising ecological validity. In some applied settings such as forensic perpetrator identification tasks, which mostly include color images, however, ecological validity might be more important than experimental control (Mayer & Ramon, 2023; Stacchi et al., 2020). Moreover, Bobak et al. (2019) showed that the use of grayscale stimuli might cause a conservative response bias, which is a tendency to make “new” face memory decisions regardless of actual face recognition ability. Therefore, presenting whole faces in color as consistent with real-life face recognition tasks might diminish conservative response bias and help to increase the ecological validity of the test.

It is necessary to assess the psychometric properties of a given face memory test to statistically evaluate its strengths and weaknesses. Investigating the psychometric properties of CFMT, Corrow et al. (2018) revealed that in the third phase of the test, which consists of face images with visual noise, the control group performed worse than participants with prosopagnosia. In other words, the third phase of the test reverses the difference between the control group and the prosopagnosics, potentially leading to a decrease in the accurate identification of prosopagnosia. In addition, Bowles et al. (2009) found that when a cutoff criterion of two *SD* was used, CFMT correctly detected only 57% of the prosopagnosic patients. Murray and Bate (2020) pointed out the same problem by showing that applying the whole or first two phases of the test resulted in almost identical hit rates (correct recognition of target faces) for prosopagnosics, which were around 60%. Moreover, they showed that the test–retest reliability of CFMT for developmental prosopagnosia was just around the acceptable psychometric standard (Spearman’s* r* = .68).

### Cambridge face memory test–long form

The Cambridge Face Memory Test–Long Form (CFMT+; Russell et al., 2009) was developed to detect super recognizers, people with extraordinary face memory. CFMT+ consists of four phases. The first three phases are identical to CFMT in terms of test structure and stimuli. The fourth phase includes 30 trials with extra noise and facial emotion expressions to increase task difficulty. Still, CFMT+ also comes with limitations, much like the original CFMT.

First, the test structure of CFMT+ may not be suitable for identifying individuals with exceptional face recognition abilities, as the test was originally designed to assess individuals at the lower end of the spectrum. Davis et al. (2020) investigated the effects of different retention intervals on face memory performance and found that a significant portion of the super recognizers, identified with a CFMT+ score of above 95, performed similarly to the control group when the test phase was not conducted immediately. This finding suggests that different retention intervals, mimicking the real-world challenge of face recognition, might be useful in identifying individuals at the high end of the spectrum. While incorporating long retention intervals between learning and test blocks could enhance the ecological validity of such tests, it also introduces significant practical challenges, such as increased administration costs and the heightened risk of data loss. Although including retention intervals might not be feasible for practical reasons, long-term retention can still be achieved by employing sequential study and test phases. However, CFMT+ uses a simultaneous study presentation in which target images are presented at the same time. Three target images of the same identity are displayed for 3 seconds in the first block of CFMT+, and six target images of different identities are presented for 20 seconds in the following blocks. Sequential study presentation contributes to transfer of working memory items to long-term memory and allows better consolidation of target items (Ricker & Cowan, 2014). Utilizing sequential study presentation may help participants learn target faces more effectively and promote long-term retention of these items.

Second, all trials in CFMT+ include target face stimuli, as the test employs a three-alternative forced-choice response type. Yet, face recognition ability requires one to correctly reject the distractor faces when the target face is absent. For example, super recognizers might be employed in the security field and could work as forensic examiners— professionals who operate in forensic and security fields following extensive training and mentoring—where they are required to recognize criminals in line-ups (Ramon et al., 2019; Robertson et al., 2016) that might not contain the criminal. Bate et al. (2018) claimed that target-absent trials are necessary to ecologically and validly evaluate face memory. They revealed that when target-absent trials were included, only 59.5% of the super recognizers identified with CFMT+ performed consistently better than the control group. Therefore, not including target-absent trials may lead to erroneous face memory assessment that may cause serious consequences in applied areas.

Third, in order to increase the difficulty of the newly added fourth phase to the CFMT+, different techniques were employed such as adding extra visual noise, including emotional facial expressions, and using the most repeated distractor face images. Even though extra visual noise added to face images might improve experimental control, it is not one of the daily challenges of face memory and might potentially decrease the ecological validity of the test. On the other hand, although including emotional expressions seems to increase ecological validity, it also carries a risk of promoting response bias (Baudouin et al., 2010). That is, smiling faces create a sense of familiarity which potentially causes a more liberal response bias to make an “old” face recognition decision (Baudouin et al., 2010). The final method for increasing the difficulty of the fourth block has been to include the most repeated distractor faces. There are 46 distractor face stimuli used in CFMT+, and 14 of the most used distractors in the first three phases of the test are presented again in the fourth phase. However, as previously mentioned (see p. 5), repeated distractor faces potentially increase participants’ liberal response bias as well, due to enhanced processing fluency for those items (e.g., Gold et al., 2007; Kramer & Berry, 2020; Kurilla, 2011). Thus, each of these methods used to increase the difficulty of CFMT+ has adverse effects on either ecological validity or response bias. We critically note that although overcoming these methodological shortcomings may diminish the potential response bias confound, it does not guarantee that it will fully eliminate it, because methodological factors are not the only source of response bias in memory performance (Baudouin et al., 2010). Moreover, these potential response bias confounds stemming from methodological factors present challenges in particular when accuracy measures cannot evaluate memory performance independently of response bias, as in CFMT or CFMT+. Therefore, in order to increase the difficulty of the test in a psychometrically valid way, the best option is to use alternative methods that consider the above methodological factors while also ensuring the use of appropriate assessment of face memory performance independently of response bias.

## University of New South Wales face test

The University of New South Wales (UNSW) Face Test was developed as a screening test for super recognizers (Dunn et al., 2020). An important advantage of the UNSW Face Test is that it comprises both face memory and face matching tasks to comprehensively evaluate face recognition ability. The face memory task includes study and test phases. In the study phase, 20 high-resolution face stimuli are sequentially presented. In the test phase, a new set of 20 target and 20 distractor face images taken from models’ social media accounts are used. To be more specific, target images in the test phase are not the exact images presented in the study phase but are different, previously unstudied photographs of the same person. Given that social media accounts often feature images with emotional expressions, this may introduce response bias as a potential confounding variable as in CFMT + (Baudouin et al., 2010).

The UNSW Face Test was initially designed to identify super recognizers, making it less effective in distinguishing between prosopagnosics and controls. Its primary objective during the test development procedure was to create a screening tool for super recognizers. Consequently, the overall performance on this test tends to be relatively low in comparison to other face memory tests (Dunn et al., 2020). Therefore, the UNSW Face Test may not be well suited for evaluating individuals at both extremes of the face memory spectrum.

### Goldsmiths unfamiliar face memory test

Goldsmiths Unfamiliar Face Memory Test (GUFMT; Jansari et al., 2020) is another test developed to address the inadequacy of existing tests in detecting super recognizers. GUFMT employs a test structure that is identical to CFMT+, with the exception of presenting each face only once to overcome the learning effect resulting from repeated exposure to target and distractor faces in CFMT+. However, GUFMT includes computer-generated animation face images which might decrease its ecological validity and has the potential to promote liberal response bias in participants compared to real human faces (Kätsyri, 2018).

GUFMT includes a section comprising a composite face task which is used to evaluate holistic face perception (see Richler et al., 2015, for further information regarding the composite face task). This section of GUFMT is based on the assumption that superior face memory is a result of holistic face perception (DeGutis et al., 2013). Indeed, research examining the relationship between superior face memory and holistic face perception often incorporates a variety of tasks aimed at assessing holistic perception (Konar et al., 2010; Richler et al., 2011; Richler & Gauthier, 2014). Despite being one of the most commonly employed holistic face perception tasks, the composite face task is subject to some structural controversies, such as the complete design versus partial design discussion (Rossion, 2013). Given that every structural decision in the composite face task can significantly influence outcomes, relying solely on this task for evaluating holistic face processing may produce unreliable results (Ross et al., 2015). Moreover, whether superior face memory is grounded in holistic processing remains a debated issue (Konar et al., 2010; Wang et al., 2012). Some studies have shown that holistic processing accounts for only a small portion of the variance in individual differences in face memory (DeGutis et al., 2013; Richler & Gauthier, 2014). This suggests that while holistic processing may play a role in face perception, its contribution to superior face memory is limited. Consequently, the reliance on the composite task within the GUFMT for identifying superior face recognizers may be problematic, given both the methodological controversies surrounding the task and the ongoing debate regarding the extent to which holistic processing contributes to individual differences in face recognition ability.

In the previous sections, the unique limitations of each face memory test have been examined under their respective subheadings. While the face memory tests discussed thus far have been widely used in the literature and possess several strengths, they also share certain areas that could benefit from further development. These tests, despite their popularity and effectiveness in various contexts, still present some common limitations that provide opportunities for refinement. In the following section, we will delve into these areas for improvement, focusing on how enhancing specific aspects could contribute to more robust face memory assessments.

## Common problems of face memory tests

### Lack of dynamic face stimuli

Although the effect of face movements on face memory is well grounded in the literature (Bennetts et al., 2015; Knight & Johnston, 1997), none of the existing face memory tests includes dynamic face stimuli. For example, widely used tests such as the CFMT, its extended version CFMT+ (Russell et al., 2009), UNSW Face Test (Dunn et al., 2020), and the GUFMT (Jansari et al., 2020) rely solely on static images during both the learning and test phases. Jenkins et al. (2011) claim that static stimuli do not consistently capture how a person looks, as there are extreme within-person variations in the static face stimuli. There are two main accounts explaining the effect of face movements on face memory: *the supplemental information hypothesis* (Knight & Johnston, 1997) and *the representation enhancement hypothesis* (Pike et al., 1997). The supplemental information hypothesis argues that face movements provide additional information. Identity-specific head movements are referred to as “dynamic identity signatures” so that these movements alone can be used to identify a person (Lander & Bruce, 2000). The representation enhancement hypothesis claims that motion provides additional viewing points which aid in forming a richer mental representation of a face. Notably, both hypotheses support the notion that face movements facilitate face memory. Therefore, including dynamic face stimuli in the face memory tests could be an important contribution to enhancing ecological validity, which, in some situations, might be more important than experimental control. For example, officers of the Metropolitan Police Force of the Greater London area, which is a police force that also includes super recognizers, are expected to encode and recognize faces from both types of stimuli (Robertson et al., 2016). By the same token, newly developed EGEFACE aims to enhance ecological validity by including both dynamic and static face stimuli.

### Methods used to increase test difficulty

Various methods have been employed to increase the difficulty of face memory tests in order to identify super recognizers, such as adding Gaussian noise to the face images, including emotional facial expressions, using the most repeated distractor faces (*CFMT+*, Russell et al., 2009), including face images from multiple races (*UNSW*, Dunn et al., 2020), and using animation faces (*GUFMT*, Jansari et al., 2020). However, most of these methods might potentially shift participants’ response bias as well. That is, like using emotional faces or the most repeated distractor faces (e.g., Baudouin et al., 2010; Gold et al., 2007; Kramer & Berry, 2020; Kurilla, 2011), using face images from multiple races has the potential to create response bias confound. For example, Slone et al. (2000) showed that participants recognized other-race faces at about the same accuracy level as own-race faces, yet they were more liberal in responding to faces from other races than to those from their own race. Consistent with the other-race effect on response bias, individuals tend to respond more liberally to artificial intelligence-generated animation faces than real human faces (Kätsyri, 2018). Therefore, these methods create a response bias problem that might be confounded with actual test performance, especially when accuracy is not measured independently of response bias.

As an alternative method, face recognition algorithms have recently been used to manipulate the block difficulty of some face matching tests (i.e., Oxford Face Matching Test, Stantic et al., 2021; Glasgow Face Matching Test 2, White et al., 2022). These algorithms calculate the similarity between target and foil faces, and since similar faces are harder to differentiate, this increases the difficulty of the test. Although this method promises to increase difficulty in an ecologically valid way, to our knowledge it has not yet been used in a face memory test. Thus, EGEFACE is the first face memory test to employ face recognition algorithms to adjust test difficulty.

### Psychometric properties requiring further investigation

Another common problem with existing tests is that the psychometric properties of these tests have not been comprehensively examined. Corrow et al. (2018) investigated the effectiveness of CFMT in detecting patients with prosopagnosia. They analyzed performance in the first two phases without visual noise, and in the third phase with visual noise, and revealed that adding visual noise to the last phase caused the performance of the control group to decrease more than that of the prosopagnosic group. Given that the inclusion of trials with visual noise in the standard CFMT has proven to be ineffective for detecting prosopagnosia, it raises concerns about the CFMT+ that incorporates an additional phase with visual noise as well. Moreover, Murray and Bate (2020) examined the test–retest reliability of CFMT, revealing a value of .68, suggesting that it does not exhibit very high reliability according to widely accepted standards (Tavakol & Dennick, 2011). It is important to highlight that not only CFMT, but also other face memory tests (Arrington et al., 2022; Dunn et al., 2020), often demonstrate psychometric properties that may not be considered exceptionally high. For example, the test–retest reliability of the UNSW Face test was also found to be .59, even lower than CFMT (Dunn et al., 2020), and some of the items in CFMT+ show a negative correlation with the total score (Arrington et al., 2022).

## Ege Face Memory Test (EGEFACE)

**Fig. 1:**
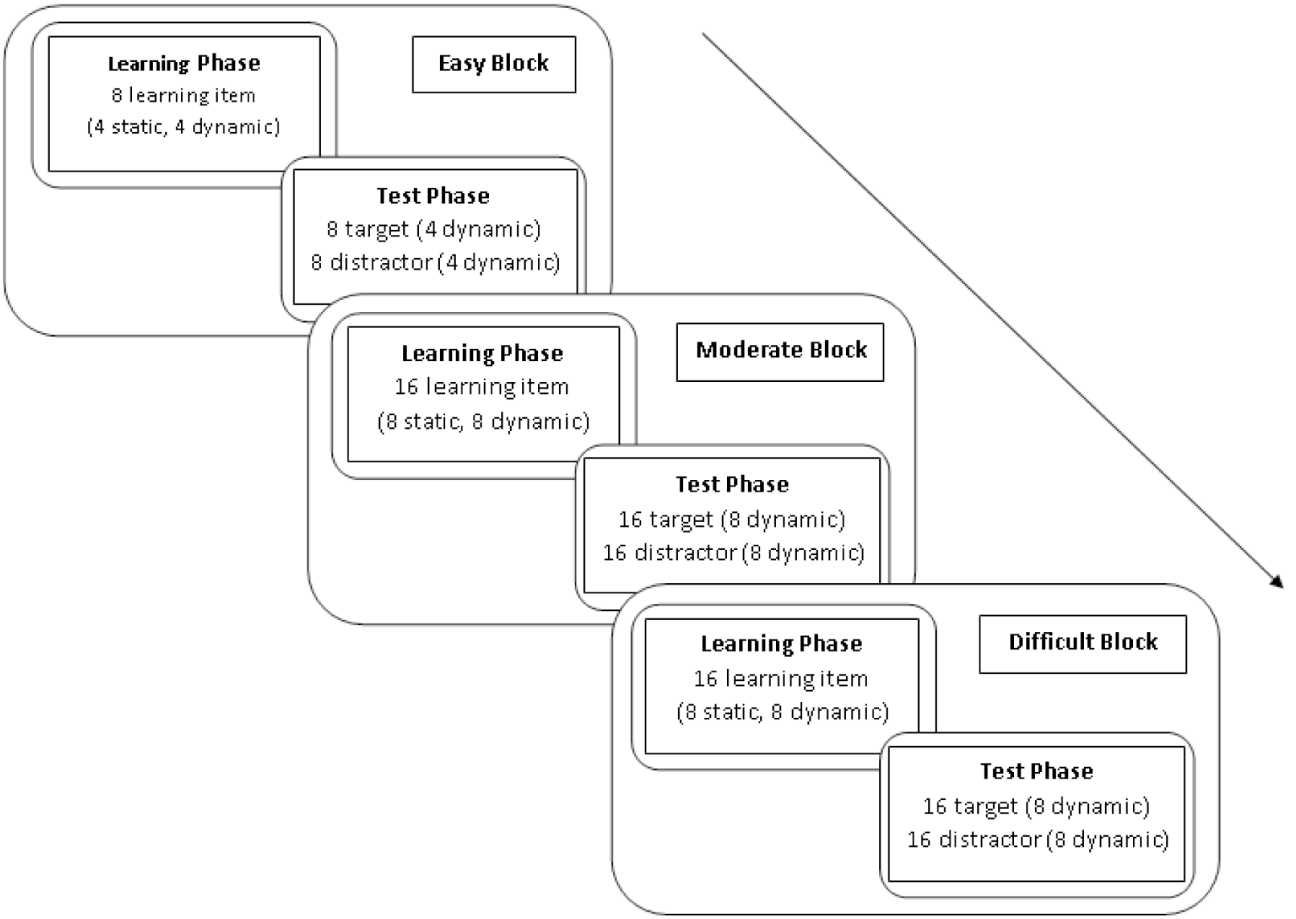
Test flow of EGEFACE

EGEFACE was developed to address existing structural and stimulus-related shortcomings of the previous face memory tests and aimed to objectively measure the full range of face recognition ability including people with prosopagnosia, those with typical ability, and super recognizers. As face memory performance differs significantly among these groups, it is essential to include separate blocks of varying difficulty. Therefore, EGEFACE consists of a total of 80 items in three blocks (16, 32, and 32 items, respectively) with increasing difficulty. Each block includes an equal number of target and foil trials (half of the items are targets and the other half are foils). The flow of EGEFACE is presented in Fig. 1. The difficulty of the blocks was adjusted using a face recognition algorithm. Each difficulty block includes a study phase and a test phase. In the study phase, target faces are presented sequentially, and participants are asked to study the faces. In the test phase, participants complete a standard old/new recognition task.

EGEFACE differs from current memory tests in a number of methodological and statistical aspects. First, we took special care to ensure ecological validity as much as we could while ensuring experimental control as well. While currently used face memory tests only include static stimuli, EGEFACE includes static and dynamic stimuli in both study and test phases so that the test reflects real-life conditions as much as possible. Our methodological approach also aims to overcome some limitations of existing tests that have the potential to promote response bias in participants’ face memory decisions, such as the repetition of target and distractor faces, using grayscale, smiling or AI-generated faces, or faces from multiple races (Bobak et al., 2019; Baudouin et al., 2010). Thus, we included only neutral, colorful, real human face stimuli from the same race and presented each of them once.

We critically note that methodological factors may diminish the potential response bias effects, yet they cannot guarantee that it will be fully eliminated, because those are not the only sources of response bias in memory performance (Bowen et al., 2020; Kapucu et al., 2008). For example, there are also remarkable individual differences in response bias, regardless of task requirements, that cannot be completely eliminated or experimentally controlled (Kantner & Lindsay, 2014). Importantly, these methodological factors present challenges in particular when accuracy measures cannot evaluate memory performance independently of response bias, as in most existing face memory tests. Indeed, current face memory tests typically measure memory performance with single-point parameters such as percent correct (Bobak et al., 2016; Davis et al., 2016). These kinds of measures have been strongly criticized for misinterpreting the effects of memory accuracy when there are also changes in response bias (Masson & Rotello, 2009). Therefore, in order to provide psychometrically valid tests that assess face memory performance independently of response bias, the use of appropriate statistical techniques is essential. To this end, in EGEFACE, recognition decisions were made on a confidence rating scale which allowed us to conduct receiver operating characteristic (ROC) analysis based on the signal detection theory (SDT; Macmillan & Creelman, 2005). By conducting the ROC analysis, we ensured that face memory accuracy could not be confounded by potential response bias effects that might stem from methodological or individual factors that cannot be controlled experimentally. When designing EGEFACE, our specific aim was to measure face memory accuracy independently of response bias, while eliminating methodological factors that have been shown to promote response bias (e.g., repetition of target and distractor faces, using grayscale, smiling or AI-generated faces, or faces from multiple races). We did not aim to measure participants’ response bias in different blocks or to compare it with previous studies; the latter is not feasible anyway, as previous tests did not report a measure of response bias.

Lastly, most of the currently used face memory tests, namely CFMT (Duchaine & Nakayama, 2006), CFMT+ (Russell et al., 2009), and GUFMT (Jansari et al., 2020), only include target-present trials. Bate et al. (2018) revealed that out of 119 people who showed superior face memory performance in multiple tests, only five individuals performed better than the control group when both target-absent and target-present trials were included. Moreover, Bobak et al. (2016) reported that when the test consisted only of target-present trials, super recognizers and the control group performed comparably. When target-absent trials were also included, the control group’s performance decreased dramatically, but super recognizers’ performance remained the same. In sum, studies point out that target-absent trials are critical for distinguishing between super recognizers and controls. Therefore, both target-absent and target-present trials are included in EGEFACE. Furthermore, the inclusion of target-absent trials also allowed us to conduct ROC analysis and examine face memory uncontaminated by potential response bias effects.

## General method

EGEFACE was developed in three stages. The *first stage* involved stimulus development, followed by the *second stage* which focused on development and testing the general structure of EGEFACE. Lastly, the *third stage* pertained to replicating the findings obtained in the previous stage. The study was approved by Ege University Research Ethics Committee.

### Stage 1: Creating a new face database

The test development stage was advertised on the social media accounts and the website of Ege Face Recognition Laboratory. A total of 101 models (53 female) aged between 18 and 35 years contributed to the construction of the face database in exchange for entry in a prize drawing for a 250₺ gift voucher. All of the models that participated in this stage of the study were of Turkish ethnic background. None of them participated in the subsequent stages.

A Logitech C920 Pro webcam was used to record 1080p-30 fps videos of the models. Adobe Premiere Pro 2020 and Adobe Photoshop 2020 were used to edit the final stimuli. Two videos of each model were recorded to be used in the study and test phases. In the study phase videos, models were asked to walk towards a fixed webcam. They were asked to look once to the right and once to the left while walking. Study phase videos ended with models looking straight into the webcam. The duration of the study phase videos was edited to be 5 seconds each. The final frames of the study phase videos in which models were looking directly at the webcam were used as static study stimuli. Importantly, no restrictions were placed on environmental cues, such as hairstyle, during the study phase, to allow participants to encode the faces in a more naturalistic and ecologically valid context.

**Fig. 2:**
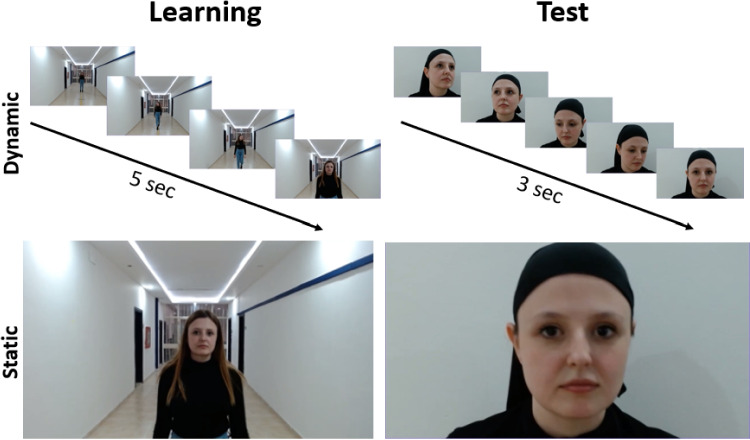
Examples of stimuli used in EGEFACE

In the test videos, models were asked to look at the corners of the square behind the camera so that their faces could be seen from different angles in the videos. Test videos ended with models looking straight into the camera. The duration of the test videos was edited to be 3 seconds each. Static test stimuli were obtained from the final frame of the test videos. In contrast to the study phase, all environmental cues, including hair, were deliberately restricted during the test phase to create a more challenging and ecologically valid scenario. This decision was based on findings from Adams et al. (2019), which showed that individuals with prosopagnosia rely heavily on environmental cues, such as hairstyle, uniform, and clothing, as compensation mechanisms. By restricting these cues in the test phase, we aimed to introduce a controlled difficulty, ensuring that participants relied solely on facial recognition without the aid of additional environmental cues. Example stimuli are shown in Fig. 2.

A face recognition algorithm (Geitgey, 2017) was used to select the stimuli that would be used in the test blocks. The face recognition algorithm, which outperforms other algorithms in terms of misdetection and achieves a 96.98% overall performance on 100,000 faces (Kabakus, 2019), can produce a distance value which is a quantitative measure of how similar/different the faces are. The algorithm works better when comparing static stimuli. To adapt it for dynamic stimuli, we used the last frame of each video as the static representation for comparison. All of the faces in the face database were compared with other same-sex faces to obtain distance values. These distance values were used to assign a given stimulus to a block. The most distant (dissimilar) faces were used in the easy block so that discriminating between the faces would be easier. In contrast, the most similar faces were assigned to the most difficult third block in order to discriminate super recognizers from neurotypical individuals. After selecting the faces for the blocks using a face recognition algorithm, half of the images were designated as target faces, while the other half were used as distractor faces. The final stimulus set was determined by a preliminary study with 10 participants. To compare the similarity of a face to other faces of the same gender within the same block across different blocks, a one-way analysis of variance (ANOVA) was conducted, with the independent variable being the block (easy, medium, difficult) and the dependent variable being the average face distance index of the face to other faces of the same gender within the same block. The results revealed a significant effect of test block on face distance index, *F*(2, 77) = 10.25, *p* < .001, *η*_p_^2^ = .21. The average distance between faces within the easy (*M* = .69, *SD* = .04) and moderate blocks (*M* = .68, *SD* = .03) is greater than the average distance between faces within the difficult block (*M* = .65, *SD* = .01), with *t*(46) = 3.54, *p* < .001, Cohen's* d* = 1.08 for the easy block and *t*(62) = 3.89, *p* < .001, Cohen's* d* = 0.97 for the moderate block. Given that an increase in face distance is expected to correspond to a decrease in average similarity, it can be inferred that the faces in the difficult block are more similar to each other. Although there is no statistically significant difference in the average face distance between the easy and medium blocks, an increase in the number of stimuli is expected to create an ecologically valid increase in block difficulty.

After the image selection for EGEFACE was completed, two data collection stages were carried out, corresponding to the *test development* and *replication* stages, respectively. The latter stage was conducted for replication purposes. The structure of EGEFACE remained the same in both of the data collection stages.

### Stage 2: Test development

#### Method

##### Participants

Participants were recruited via announcements on the social media accounts and the website of Ege Face Recognition Laboratory. A total of 113 individuals (86 female) aged between 18 and 30 years participated in the study. Participants were asked to complete the EGEFACE, CFMT+, and KFMT. All of the participants completed EGEFACE. However, for various reasons, five participants did not complete the CFMT+, so 108 participants (83 female) finished both EGEFACE and CFMT+. Additionally, 13 participants did not complete the KFMT, resulting in 100 participants (75 female) completing both EGEFACE and KFMT.

##### Materials. Ege Face Memory Test

The test comprises 80 items distributed across three blocks, with 16, 32, and 32 items in each block, respectively. Each block contains an equal number of target and foil trials, ensuring that half of the items are targets and the other half are foils. The test includes three blocks of increasing difficulty, which are always completed in the same order, progressing from easy to difficult. Importantly, each model is presented only once during the test. If a model's face is used as a target or foil in one block, it does not appear in other blocks, ensuring that targets and foils do not repeat across blocks. During the study phases, face stimuli are presented sequentially, for 5 seconds each, following a fixed order. In the subsequent test phases, test stimuli are also presented in a fixed order for 3 seconds each and participants are asked to decide whether they recognize them from the preceding study phase by making an old/new decision on a six-point confidence rating scale (1: “sure old”, 6: “sure new”). Confidence ratings were used to conduct ROC analysis based on the SDT (Macmillan & Creelman, 2005). We provide detailed information in the “Statistical analyses” section regarding how ROC analysis was performed to ensure measurement of participants’ face memory sensitivity independently from their response bias for making “old/new” judgments.

##### Cambridge face memory test–long form

The test consists of four phases and 102 trials. CFMT+ involves learning six unfamiliar male faces and subsequently assessing the recognition of these faces in a three-alternative forced-choice task. The test structure and stimuli are discussed in detail above. Performance in CFMT+ is measured by the percentage of correctly recognized items.

##### Kent face matching test–short form

Even though the Oxford Face Matching Test (OFMT Stantic et al., 2021) and Glasgow Face Matching Test 2 (White et al., 2022) are currently gaining traction, the Glasgow Face Matching Test (GFMT) and KFMT were the most widely used face matching tests at the time this study was designed. The GFMT uses grayscale stimuli, and participants typically demonstrate very high performance. However, KFMT was chosen for our study due to several distinct advantages. Firstly, the KFMT employs colored stimuli, which more accurately reflect real-world conditions. The photographs used in the KFMT have an average interval of 8 months between them, which introduces natural variations in appearance and further enhances the ecological validity of the test. Unlike the GFMT, where external features such as hair and clothing are removed, the KFMT retains these external features. This retention is crucial because it mirrors the everyday task of face matching, where external features play a significant role. Moreover, the KFMT has been shown to better capture the distribution of face matching performance across a wide range of participants (Fysh & Bindemann, 2018). The test–retest reliability of the KFMT has been reported as .67, and it has a correlation of .45 with the GFMT. These statistics suggest that while both tests measure a similar cognitive construct related to face matching, the KFMT offers a more ecologically valid assessment. Given its higher ecological validity and better representation of face matching performance, KFMT was selected for use in our study. This selection aligns with our goal of developing a test that accurately reflects real-world face recognition scenarios and ensures the ecological validity of the EGEFACE.

The test consists of 40 trials (20 matches, 20 mismatches). On each trial, two face stimuli are presented simultaneously, and participants are asked whether the two face images belong to the same person. Each image pair in KFMT consists of two photographs: One is a carefully controlled high-resolution target photograph, similar to a passport picture, capturing individuals in ideal conditions. The other is an ambient photograph, such as a student ID picture, where the individuals are portrayed in various poses, with diverse facial expressions, providing a more natural and varied representation.

##### Procedure

After providing informed consent and demographic information, participants were asked to complete EGEFACE, CFMT+, and KFMT. The order of the tests was counterbalanced across participants. All tests were applied in the Ege Face Recognition Laboratory via testable.org (Rezlescu et al., 2020). The participants were given instructions for all tests before starting the respective test.

Before completing EGEFACE, participants were instructed that the EGEFACE test would require them to first learn a series of faces and then differentiate these learned faces from new ones. The test was divided into three distinct blocks, each comprising a learning phase followed by a testing phase. During the learning phase, participants were instructed to carefully observe and memorize the faces presented to them in sequence. In the subsequent testing phase, they were shown both the previously learned faces and new faces and were required to identify the learned faces. To express their confidence in distinguishing old faces from new ones, participants used a six-point scale ranging from 1 to 6. This scale was prominently displayed at the bottom of the screen during all test trials to ensure that participants could easily refer to it. Before beginning the actual test, participants underwent a practice session to become familiar with the test procedures. In this session, cartoon faces were presented during the learning phase, and participants were later asked to distinguish these faces from others in the testing phase.

For the CFMT+ test, participants were first instructed to carefully study the target faces presented to them simultaneously during the learning phase. Immediately after, they were presented with three faces and asked to identify the target face they had just learned by selecting the corresponding key on the keyboard.

For the KFMT, participants were instructed to examine two faces presented side by side and determine whether they depicted the same person or two different individuals. They were asked to make this decision as accurately and quickly as possible by selecting the appropriate response on the keyboard.

### Statistical analyses

In order to evaluate the EGEFACE test performance uncontaminated by response bias effects, we conducted ROC analysis based on the SDT (Macmillan & Creelman, 2005; Rotello et al., 2008). Participants’ confidence judgments were used to create the ROC curves that plot hit rates against false alarm rates at each confidence level, providing a graphical representation of a participant’s performance across various old/new decision thresholds. To evaluate participants’ test performance, the area under the ROC curve (AUC) parameter was estimated based on each difficulty block, which quantifies participants’ ability to discriminate between previously presented faces and new ones at each difficulty level of EGEFACE. We preferred to use AUC over *d′* (one of the conventional measures of accuracy) as our main dependent measure because it provides a nonparametric measure of memory sensitivity without any model assumptions.^1^ AUC ranges from 0 to 1, with 0.5 indicating performance at a chance level and 1 indicating perfect discrimination ability. Thus, the higher the AUC value, the better the performance in distinguishing between old faces and new faces.

Along with mean AUC values, we also provided conventional values of accuracy—percentage correct—for target-present, target-absent, and block total performance, as well as overall test accuracy, and distribution parameters of skewness and kurtosis.

#### Results

##### Block difficulty

Descriptive statistics for accuracy (percentage correct) for target-present, target-absent, block total, and AUC across easy, moderate, and difficult blocks, as well as the overall test performance, are presented in Table 1. Skewness and kurtosis values are also included to assess the distribution of these parameters. To examine the impact of block difficulty on test performance during the test development stage, we conducted a one-way ANOVA with test difficulty as the three-level within-subject independent variable (easy, moderate, difficult). The dependent variable was the participants’ performance as measured by the AUC in the separate test blocks. The results revealed a significant effect of block difficulty on test performance, with the assumption of sphericity met, *F*(2, 224) = 268.32, *p* < .001, *η*_p_^2^ = .71. Participants exhibited their best performance in the easy block (*M* = .88, *SD* = .10) and their worst in the difficult block (*M* = .61, *SD* = .11), as expected, which is consistent with the results from the 10-participant preliminary study. This pattern of results indicated a consistent decline in performance as block difficulty increased (Table 2), confirming the successful manipulation of block difficulty using a face recognition algorithm. Overall, the mean test performance closely resembled that of the moderate block (*M = *.73 *SD* = .09). Most participants achieved nearly flawless performance in the easy block, while their performance notably deteriorated in the difficult block. Additionally, all of the difficulty blocks showed a low, but significant, positive correlation with CFMT+ scores (ranging from *r* = .22 to *r* = .32, *p* < .05). These results indicate that while participants' performance in the face recognition task declined with increasing block difficulty, their scores on CFMT+ still maintained a weak positive relationship across all levels of difficulty. Correlations with both CFMT+ and KFMT are detailed in the supplementary materials, available for further inspection on OSF.

**Table 1:** Descriptive statistics for test performance across blocks in the test development stage

	Easy block				Moderate block				Difficult block				EGEFACE total			
	Target-present	Target-absent	Block total	AUC	Target-present	Target-absent	Block total	AUC	Target-present	Target-absent	Block total	AUC	Target-present	Target-absent	Block total	AUC
Valid	113	113	113	113	113	113	113	113	113	113	113	113	113	113	113	113
Missing	0	0	0	0	0	0	0	0	0	0	0	0	0	0	0	0
Mean	80.64	82.97	81.80	0.88	65.27	70.85	68.06	0.73	54.04	62.00	58.02	0.61	63.85	69.74	66.79	0.72
SD	17.28	13.88	10.38	0.10	13.52	14.93	7.81	0.09	17.12	17.38	9.43	0.11	12.15	12.23	5.87	0.70
Skewness	−1.06	−0.99	−0.50	−0.80	0.02	−0.83	−0.50	−0.15	0.02	−0.24	−0.16	−0.08	0.19	−0.60	0.27	0.14
SE of skewness	0.23	0.23	0.23	0.23	0.23	0.23	0.23	0.23	0.23	0.23	0.23	0.23	0.23	0.23	0.23	0.23
Kurtosis	1.63	2.08	−0.17	0.46	−0.36	1.00	0.28	−0.09	−0.02	−0.29	−0.55	−0.24	0.07	0.75	−0.41	0.87
SE of kurtosis	0.45	0.45	0.45	0.45	0.45	0.45	0.45	0.45	0.45	0.45	0.45	0.45	0.45	0.45	0.45	0.45
Minimum	12.50	25.00	50.00	0.54	31.25	18.75	46.88	0.51	12.50	12.50	34.38	0.33	35.00	27.50	53.75	0.49
Maximum	100.00	100.00	100.00	1.00	93.75	100.00	84.38	0.93	100.00	100.00	78.13	0.90	95.00	95.00	80.00	0.92

Descriptive statistics for accuracy (percentage correct) for target-present and target-absent trials and for the total block in the test development stage. AUC values also present test performance for each block uncontaminated by response bias effects

**Table 2:** Pairwise comparisons for AUC across difficulty blocks in the test development stage

			Mean difference	SE	*t*	Cohen’s *d*	*p*_bonf_
Easy	vs.	Moderate	0.15	0.01	13.12	1.54	< .001
Easy	vs.	Difficult	0.26	0.01	23.10	2.70	< .001
Moderate	vs.	Difficult	0.11	0.01	9.98	1.17	< .001

*SE* = standard error of the mean differences

##### Reliability

In assessing the reliability of EGEFACE, we examined internal consistency and split-half reliability for both target-present and target-absent items. The split-half reliability estimates and internal consistency were calculated separately for all target-present and target-absent trials across the entire test. This means that the analyses were not conducted within individual blocks, but instead, trials from the entire test were combined and analyzed together. By doing so, we aimed to assess the overall reliability of the test as a whole, rather than focusing on the reliability of each block independently. For target-present items during the test development stage, the split-half reliability analysis yielded a coefficient of .77, demonstrating the reliability of these items. Furthermore, we evaluated the internal consistency of the target-present items, resulting in a Cronbach’s alpha value of *α* = 0.76. Importantly, no individual item removal would have significantly affected Cronbach’s alpha during the test development stage. The corrected item–total score, a measure of internal consistency, was calculated by correlating each item's score with the total score derived from the remaining items, excluding the item in question. This method, commonly used in psychometric evaluations to identify items that may not align well with the overall construct (Nunnally & Bernstein, 1994; Field, 2013), provided additional insights into item reliability, with correlations ranging from *r* = .04 to *r* = .42.

For the target-absent items in the test development stage, the split-half reliability analysis yielded a coefficient of .70, indicating the reliability of these items as well. The internal consistency among the target-absent items was *α* = .79, and like the target-present items, no specific item removal would have notably influenced internal consistency. The corrected correlations between individual items and the total score ranged from *r* = .07 to *r* = .45.

##### Relationships between CFMT+, KFMT, and EGEFACE scores

To better understand how EGEFACE aligns with established measures of face recognition, we first examined its relationship with CFMT+, a widely used test of face memory. To reiterate, test performance is measured by the AUC parameter in EGEFACE, and by percentage correct in CFMT+.

In the test development stage, 108 participants^2^ completed both EGEFACE and CFMT+. EGEFACE scores ranged between .49 and .92, with a mean score of .72 (*SD* = .07), and CFMT+ scores ranged between 45 and 96, with a mean score of 72.06 (*SD* = 11.38). Our analysis revealed a statistically significant moderate positive correlation between EGEFACE and CFMT+, *r*(107) = .37, *p* < .001. This moderate correlation persisted despite structural and stimulus-type differences between the tests. Considering that other face memory tests with similar structures to EGEFACE (Jansari et al., 2020; Dunn et al., 2020) have reported similar correlations (from .30 to .52) with CFMT+, these findings indicate that EGEFACE and CFMT+ assess a similar cognitive skill.

To further examine EGEFACE’s relationship with face matching performance, we analyzed its correlation with KFMT, a face matching test. EGEFACE scores ranged between .49 and .92, with a mean score of .72 (*SD* = .07), and KFMT scores ranged between 50 and 90, with a mean score of 67.90 (*SD* = 8.78). In the test development stage, involving 100 participants who completed both tests, we found a small but statistically significant positive correlation between the EGEFACE and KFMT, *r*(100) = .25, *p* < .05.

##### Item type

EGEFACE consists of three blocks of study and test phases, with both phases including static and dynamic stimuli. Therefore, there are four types of target items in the test: those that are (i) learned with dynamic stimuli and tested with dynamic stimuli, (ii) learned with dynamic stimuli and tested with static stimuli, (iii) learned with static stimuli and tested with dynamic stimuli, and (iv) learned with static stimuli and tested with static stimuli. Other face memory tests consist of only static learning and static test stimuli. The effect of the item type was examined in the test development stage. A one-way within-subject ANOVA was conducted to examine the effect of item type on the performance for the target items. The independent variable was the target item type (dynamic-dynamic, dynamic-static, static-dynamic, and static-static) and the dependent variable was the hit rate for target items. The hit rate was calculated by collapsing the confidence ratings from the six-point confidence scale; if a participant indicated that they recognized a target face by selecting 1, 2, or 3 on the scale, it was counted as a hit. The total number of hits for the relevant item type was then divided by the total number of target faces of the corresponding item type to determine the hit rate. The results revealed that target item type significantly affected test performance, with the assumption of sphericity met, *F*(3, 336) = 12.55, *p < *.001, *η*_p_^2^ = .10. Participants performed best for the items that were congruently presented: performance was significantly better for both dynamic-dynamic target items (*M* = .70, *SD* = .19) and static-static target items (*M* = .66, *SD* = .16) than for dynamic-static (*M* = .60, *SD* = .16) and static-dynamic target items (*M* = .60, *SD* = .18). Pairwise comparisons are presented in Table 3. Moreover, dynamic-static and static-dynamic item types showed low positive correlations with the CFMT+, indicating some shared variance with face recognition abilities. Additional correlation analyses with other tests are available in the supplementary materials.

**Table 3:** Pairwise comparisons for hit rate across target types in the test development stage

			Mean difference	SE	*t*	Cohen's *d*	*p*_bonf_
Dynamic-dynamic	vs.	Dynamic-static	0.10	0.02	4.99	0.55	< .001
Dynamic-dynamic	vs.	Static-dynamic	0.10	0.02	5.22	0.58	< .001
Dynamic-dynamic	vs.	Static-static	0.04	0.02	2.04	0.23	0.25
Dynamic-static	vs.	Static-dynamic	0.01	0.02	0.23	0.03	1.00
Dynamic-static	vs.	Static-static	−0.06	0.02	−2.95	−0.33	0.02
Static-dynamic	vs.	Static-static	−0.06	0.02	−3.18	−0.35	0.01

*SE* = standard error of the mean differences

#### Discussion

The findings obtained at the test development stage indicate that the development of EGEFACE has been successfully completed. The next stage is the replication stage, which is designed to replicate and validate the findings obtained in the test development stage.

### Stage 3: Replication

#### Method

##### Participants

To increase the diversity of our participants for the replication stage, announcements for data collection for EGEFACE were extended to the İzmir Bakırçay University and the Pamukkale University, in addition to Ege University students, as well as the social media accounts and the website of the Ege Face Recognition Laboratory. Participants from Ege University completed CFMT+ online and EGEFACE and KFMT in the laboratory, whereas participants from different universities completed all of the tests online. A total of 589 individuals participated in the study. One participant who completed the tests online was excluded from the analysis due to an apparent misunderstanding of the task, resulting in a final sample size of 589 participants. Out of 589 individuals, 319 (213 female) participated in the replication stage in the laboratory, and 270 (208 female) participants completed the tests online. The mean age was 23.51 (*SD = *7.75) for laboratory participants, and 20.97 (*SD = *4.58) for online participants.

##### Materials

The materials were identical to the test development stage, in which EGEFACE, CFMT+, and KFMT were used.

##### Procedure

All participants provided informed consent and demographic information. They were asked to complete EGEFACE, CFMT+, and KFMT. Individuals who responded to the advertisement were sent a link to complete CFMT+ online. After the completion of CFMT+, participants were invited to the laboratory to complete EGEFACE and KFMT. If participants were not able to participate in the laboratory, they were asked to complete the tests online. The order of the tests was fixed, as participants completed tests in the following order CFMT+, EGEFACE, KFMT. The instructions for the tests were the same as for stage 2.

#### Results

##### Descriptive data for EGEFACE

**Fig. 3:**
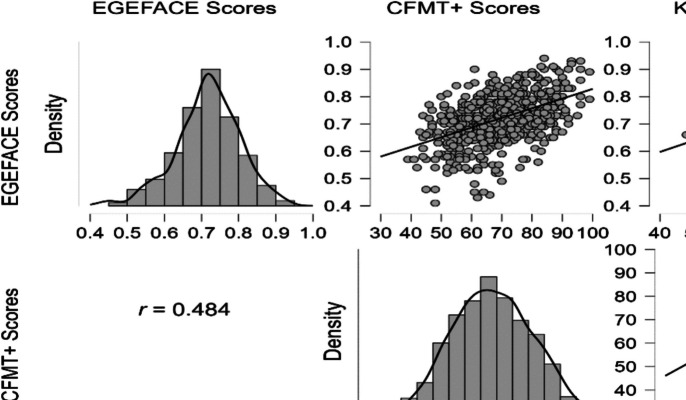
Performance distributions and correlations between EGEFACE, CFMT+, and KFMT

The data collected from the 589 participants are reported in Table 4 and visualized in Fig. 3. On average, participants correctly identified 67.12% of the items, with a standard deviation of 7.76% (AUC = 0.72 ± 0.09). The distribution of the AUC scores approximates a normal distribution, with representation across both extremes of performance. Specifically, 11 participants scored above (+2 *SD*) and 17 participants scored below (−2 *SD*) the mean. This distributional pattern suggests that the test is sensitive to variations in face recognition ability across the spectrum, supporting its applicability for assessing both high and low performers.

**Table 4:** Descriptive statistics for test performance across blocks in the replication stage

	Easy block				Moderate block				Difficult block				EGEFACE total			
	Target-present	Target-absent	Block total	AUC	Target-present	Target-absent	Block total	AUC	Target-present	Target-absent	Block total	AUC	Target-present	Target-absent	Block total	AUC
Valid	589	589	589	589	589	589	589	589	589	589	589	589	589	589	589	589
Missing	0	0	0	0	0	0	0	0	0	0	0	0	0	0	0	0
Mean	79.48	83.38	81.43	0.87	64.62	71.25	67.94	0.73	54.08	64.24	59.16	0.61	63.37	70.87	67.12	0.72
SD	18.25	15.72	12.46	0.12	15.59	16.35	9.51	0.11	16.66	18.01	10.37	0.12	12.88	13.63	7.76	0.09
Skewness	−1.09	−1.36	−1.14	−1.58	−0.30	−0.53	−0.52	−0.75	−0.06	−0.29	0.25	0.12	−0.35	−0.43	−0.20	−0.40
SE of skewness	0.10	0.10	0.10	0.10	0.10	0.10	0.10	0.10	0.10	0.10	0.10	0.10	0.10	0.10	0.10	0.10
Kurtosis	1.35	2.87	2.36	3.53	0.03	0.50	1.29	1.50	−0.20	−0.13	−0.15	−0.18	0.13	0.23	0.31	0.56
SE of kurtosis	0.20	0.20	.20	.20	0.20	0.20	0.20	0.20	0.20	0.20	0.20	0.20	0.20	0.20	0.20	0.20
Minimum	0.00	0.00	12.50	0.31	6.25	12.50	21.88	0.22	6.25	0.00	34.38	0.31	15.00	15.00	43.75	0.41
Maximum	100.00	100.00	100.00	1.00	100.00	100.00	90.63	1.00	100.00	100.00	93.75	0.96	97.50	97.50	90.00	0.94

Descriptive statistics for accuracy (percentage correct) for target-present and target-absent trials and for the total block in the replication stage. AUC values also present test performance for each block uncontaminated by response bias effects

##### Block difficulty

Descriptive statistics for accuracy (percentage correct) for target-present, target-absent, block total, and AUC across easy, moderate, and difficult blocks, as well as the overall test performance, are presented in Table 4. This table provides a comprehensive overview of the performance metrics collected throughout the study. To further evaluate the distribution of these parameters, skewness and kurtosis values are also included**.**

**Fig. 4:**
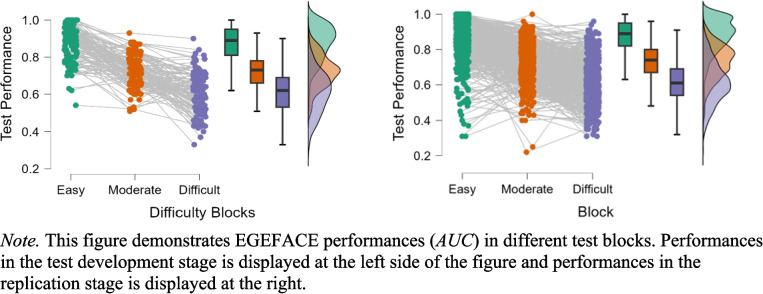
EGEFACE difficulty block performance. *Note.* This figure demonstrates EGEFACE performance (AUC) in different test blocks. Performance in the test development stage is displayed on the left side of the figure, and performance in the replication stage is displayed on the right

In the replication stage, the impact of block difficulty on test performance was reevaluated. A one-way analysis of variance (ANOVA) with block difficulty as an independent within-subject variable (easy, moderate, difficult) revealed a significant effect of block difficulty, with Greenhouse–Geisser correction applied due to the violation of the assumption of sphericity, *F*(1.95, 1146.61) = 1141.41, *p* < .001, *η*_p_^2^ = .66. In both the test development and replication stages, participants demonstrated their highest performance in the easy block and their lowest in the difficult block, thereby reconfirming the progressive increase in block difficulty (Fig. 4). This consistent decline in performance throughout the three difficulty levels provided additional evidence for the successful manipulation of block difficulty, achieved through a face recognition algorithm and a 10-participant preliminary study. Notably, the mean overall test performance (*M* = .72, *SD* = .09) closely approximated that of the moderate block. In the easy block, most participants achieved nearly perfect performance (*M* = .87, *SD* = .12), while their performance decreased noticeably in both the moderate block (*M* = .73, *SD* = .10) and difficult block (*M* = .61, *SD* = .12). All pairwise comparisons were significant (Table 5). Therefore, the replication stage confirmed that with increasing difficulty of test blocks, EGEFACE may be used as an alternative tool to evaluate face memory abilities. Moreover, the analysis revealed that the difficulty blocks (easy, moderate, difficult) all showed a low but significant positive correlation with CFMT+ (*r* values ranging between .34 and .39, *p* < .001), indicating that test difficulty levels were similarly related to the CFMT+. The correlations between block difficulty and KFMT were also comparable (*r* values ranging between .28 and .38, *p* < .001). All correlation analyses between block difficulty, CFMT+, and KFMT are available in supplementary materials for further inspection on OSF.

**Table 5:** Pairwise comparisons for AUC across difficulty blocks in the replication stage

			Mean difference	SE	*t*	Cohen's *d*	*p*_bonf_
Easy	vs.	Moderate	0.14	0.01	26.28	1.22	< .001
Easy	vs.	Difficult	0.26	0.01	47.91	2.23	< .001
Moderate	vs.	Difficult	0.12	0.01	21.63	1.01	< .001

*SE* = standard error of the mean differences

##### Reliability

We conducted separate analyses for target-present and target-absent items to assess the reliability of EGEFACE at this stage.

The split-half reliability analysis for target-present items improved to a coefficient of .72 during this stage. We also examined the internal consistency, resulting in a Cronbach’s alpha value of *α* = .78. As in the test development stage, no individual item removal would have significantly increased Cronbach’s alpha in the replication stage. The corrected item–total score correlations ranged from *r* = .17 to *r* = .34, providing additional insights into item reliability.

The split-half reliability analysis yielded a coefficient of .78, indicating the reliability of these items in the replication stage. The internal consistency among the target-absent items was α = .85, and like the target-present items, no specific item removal would have notably increased internal consistency. The corrected correlations between individual items and the total score ranged from *r = *.18 to* r* = .43.

Overall, the reliability analyses conducted for both target-present and target-absent items replicated the reliability of these items achieved in the test development stage.

##### Relationships between CFMT+, KFMT, and EGEFACE scores

We investigated the relationship between EGEFACE and previously developed tests of face recognition (CFMT+; Russell et al., 2009) and face perception (KFMT; Fysh & Bindemann, 2018). Using 589 participants who completed both the EGEFACE and CFMT+ in the replication stage, once again, a statistically significant moderate positive correlation was observed between EGEFACE and CFMT+, *r*(589) = .48, *p* < .001. This consistent finding across the two stages reconfirmed the moderate correlation between the two tests, suggesting that EGEFACE and CFMT+ indeed measure a similar cognitive skill.

We reexamined the relationship between EGEFACE and KFMT with 301 participants, and the correlation between EGEFACE and KFMT was moderate, *r*(301) = .43, *p* < .001, lower than CFMT+ as expected. Additionally, it is worth noting that the correlations between other newly developed face memory tests (Dunn et al., 2020; Jansari et al., 2020) and face matching tests (Burton et al., 2010; Fysh & Bindemann, 2018) also fell within the small to moderate range, further supporting the distinction between these cognitive skills (Fig. 4).

##### Analysis of high and low performers on EGEFACE

**Fig. 5:**
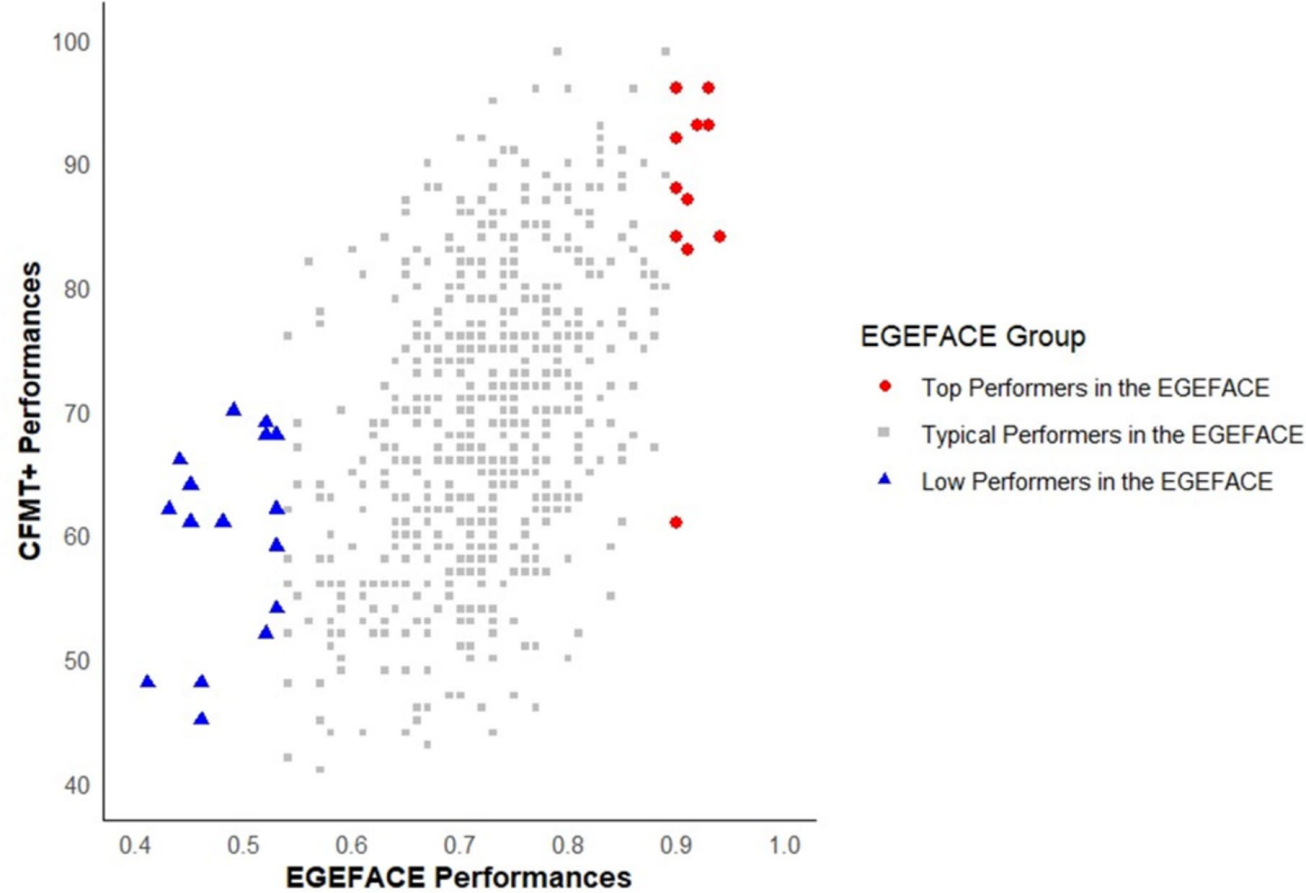
CFMT+ performance of low, typical, and high performers in EGEFACE

To assess the sensitivity of EGEFACE, participants were divided into three groups based on their performance: low performers (below –2 *SD*), typical performers (within ±2 *SD*), and high performers (above +2 *SD*). These groups were further analyzed based on their CFMT+ scores to explore the consistency across tests. Figure 5 illustrates the relationship between CFMT+ scores and EGEFACE scores for low, typical, and high performers as identified by EGEFACE.

As can be seen in Table 6, among the high performers in EGEFACE, the mean CFMT+ score was 87.00 (*SD* = 9.85), a score that approaches the range typically associated with super recognizers, often defined as scoring between 85 and 90 (Bobak et al., 2016; Ramon, 2021). This suggests that many individuals in this high-performing group possess an advanced level of face recognition ability. Notably, all but one participant in this group scored at elevated levels on the CFMT+, demonstrating consistent face recognition skills. Within this subset, five participants achieved scores above 90, which is generally considered a strong indicator of super recognizer-level performance, reflecting a robust and highly accurate ability to remember and distinguish faces.

**Table 6:** Descriptive statistics of CFMT+ scores across EGEFACE performance groups

	Low group	Typical group	High group
Valid	17	561	11
Mean	59.94	68.74	87.00
SD	7.86	11.73	9.85
Minimum	45.00	39.00	61.00
Maximum	70.00	99.00	96.00

For the low performers in EGEFACE, the mean CFMT+ score was 59.4 (*SD* = 7.86). While this score does not meet the conventional cutoff for prosopagnosia (ranging from −1.7 *SD* to −2 *SD* below the mean; Barton & Corrow, 2016; DeGutis et al., 2014), it suggests relatively low face recognition ability. While the mean CFMT+ score of the low-performing EGEFACE group was relatively low, only a small subset (3 of 17) scored within a range that may be associated with prosopagnosic performance. This limited overlap suggests that while EGEFACE is sensitive to variations in face recognition performance, it demonstrates only low-level consistency with CFMT+ for detecting prosopagnosic-level deficits.

##### Item type

The effect of the target item type on hit rate was examined in the replication stage. A one-way within-subject ANOVA was conducted to examine the effect of item type on performance for the target items. The independent variable was the target item type (dynamic-dynamic, dynamic-static, static-dynamic, static-static), and the dependent variable was the hit rate for target items. As the assumption of sphericity was violated, the Greenhouse–Geisser correction was applied. The results revealed that target item type significantly affected performance, *F*(2.94, 1725.64) = 92.42, *p* < .001, *η*_p_^2^ = .14. Pairwise comparisons are presented in Table 7. Participants performed best for the items that were congruently learned and tested as dynamic (*M* = .70, *SD* = .17). Performance for the items that were learned and tested as static (*M* = .66, *SD* = .18) was significantly higher than for both dynamic-static (*M* = .61, *SD* = .18) and static-dynamic (*M* = .57, *SD* = .19). The lowest performance was for the items that were learned static and tested with dynamic stimuli (*M* = .57, *SD* = .18). In addition, analysis of target types revealed low but significant positive correlations between all target types and CFMT+ (*r* values ranging between .09 and .24, *p* < .05). Notably, the static-static target type exhibited the highest correlation with CFMT+, suggesting that participants who performed better on the static-static trials may have relied on facial recognition skills that overlap with those measured by the CFMT+. Since both tests involve static facial images, the ability to encode and recognize these types of stimuli could be more aligned between the two tasks, potentially explaining the higher, though still low-level, correlation observed compared to other item types involving dynamic stimuli. Correlations between item type, CFMT+, and KFMT are provided in supplementary materials on OSF for further inspection.

**Table 7:** Pairwise comparisons for hit rate across target types in the replication stage

			Mean difference	SE	*t*	Cohen's *d*	*p*_bonf_
Dynamic-dynamic	vs.	Dynamic-static	0.09	0.01	11.18	0.52	< .001
Dynamic-dynamic	vs.	Static-dynamic	0.13	0.01	15.40	0.72	< .001
Dynamic-dynamic	vs.	Static-static	0.04	0.01	4.87	0.23	< .001
Dynamic-static	vs.	Static-dynamic	0.04	0.01	4.23	0.20	< .001
Dynamic-static	vs.	Static-static	−0.05	0.01	−6.31	−0.29	< .001
Static-dynamic	vs.	Static-static	−0.09	0.01	−10.54	−0.49	< .001

*SE* = standard error of the mean differences

#### Discussion

The findings in the replication stage revealed that the results of the test development stage were generally replicated. The test development and replication stages were successfully completed. Further results will be discussed in the general discussion section.

## General discussion

In the present study, we first developed EGEFACE, creating a new dynamic and static face database to overcome the shortcomings of existing face memory tests, and then examined its psychometric properties in two stages, test development and replication, in separate samples. To further investigate the test’s sensitivity, during the replication stage only, we compared CFMT+ scores of EGEFACE’s high and low performers (+2 SD). Through the incorporation of ecologically valid difficulty blocks and the utilization of both dynamic and static stimuli, this novel face memory test offers researchers an alternative means of assessing face memory abilities.

One of the important properties of EGEFACE is that the difficulty blocks were created via a face recognition algorithm. To increase test difficulty, the commonly used face memory tests employ methods such as repeating target and distractor faces, adding visual noise, and using animation faces (Russell et al., 2009; Jansari et al., 2020). However, the methods employed may not reflect everyday face memory challenges, or their potential to promote response bias. EGEFACE is the first face memory test that employs a face recognition algorithm to increase test difficulty. The results from the test development and replication stages confirmed that the difficulty of the test blocks gradually increases. EGEFACE was designed with the inclusion of various difficulty blocks, aiming to provide an alternative face memory test to evaluate a wide face memory spectrum.

Importantly, EGEFACE exhibits similar levels of psychometric properties, with arguably higher internal consistency, relative to other face memory tests; however, it achieves these properties alongside a strong argument for high ecological validity. To assess the psychometric properties of EGEFACE, internal consistency and split-half reliability were investigated. Reliability analysis conducted for target-present and target-absent items confirmed the reliability of target-present items as well as that of the target-absent items. EGEFACE demonstrated high split-half reliability for both target-present and target-absent items. The assessment of internal consistency and split-half reliability provided compelling evidence of EGEFACE's reliability as a face memory test. Convergent validity was examined to investigate the validity of the test. EGEFACE demonstrated a moderate positive correlation with CMFT+. Regarding the stimulus type and structural differences between tests, the moderate correlation indicates that these tests evaluate similar cognitive skills. Moreover, the correlation between EGEFACE and KFMT was low to moderate, as face memory and face matching skills are related but separate abilities (Bobak et al., 2016). In contrast to face memory, which involves both face perception and memory components, face matching only contains a face perception component (Fysh, 2018). Even though a stronger correlation between CFMT+ and EGEFACE was expected to confirm the convergent validity of the test, a moderate correlation between face memory tests was observed. Differences in the structure of the tests, performance measurement methods, and stimulus characteristics may have caused the observed correlation to remain at this level.

Notably, the sensitivity of EGEFACE was examined through comparisons of CFMT+ scores of high and low performers’ (±*2 SD*) on EGEFACE. The findings offer preliminary evidence regarding the sensitivity of EGEFACE in differentiating levels of face recognition ability and its potential alignment with established measures like CFMT+. Results indicate a stronger correspondence between EGEFACE and CFMT+ scores within the high-performing group, whose scores on CFMT+ approached ranges typically associated with super recognizers. This suggests that EGEFACE may represent a promising tool for evaluating individuals with heightened face recognition abilities. In contrast, alignment between the two measures was less pronounced in the low-performing group, with only three individuals (3 of 17) scoring within the range often associated with prosopagnosic performance. This discrepancy implies that while EGEFACE effectively captures a range of face recognition abilities, it may lack precision in identifying prosopagnosic-level deficits as defined solely by CFMT+ performance.

The variability in consistency, particularly in the low-performing group, may reflect ongoing challenges in defining cutoff scores for prosopagnosia. The literature suggests that accurately identifying prosopagnosia often requires multiple assessment tools, including objective face recognition tests and self-reported difficulties in everyday face recognition (DeGutis et al., 2023). Thus, while EGEFACE demonstrates promise as a sensitive measure, it should be used in conjunction with additional assessments for a comprehensive evaluation of face recognition abilities at the lower end of the spectrum.

The findings support a moderate alignment between EGEFACE and CFMT+ across different performance levels, emphasizing EGEFACE’s utility as a sensitive measure of face recognition ability. Nonetheless, the findings highlight the need for cautious interpretation, particularly in identifying super recognizers or prosopagnosics based solely on EGEFACE scores. Future research with larger, demographically balanced samples and multi-method assessments is encouraged to further validate these preliminary associations and clarify EGEFACE's role as a diagnostic or screening tool.

Another innovative feature of EGEFACE is that the test consists of both static and dynamic stimuli. Even though some important efforts have been made in developing new face memory tests that are better able to detect super recognizers and prosopagnosics (Dunn et al., 2020; Jansari et al., 2020; Arrington et al., 2022), none of these tests includes dynamic stimuli. Consistent with the current literature that movement in faces affects face memory (Bennetts et al., 2013; Xiao et al., 2014), the participants in our study performed best for dynamic learned and dynamic tested target items. The better performance for dynamic learned/dynamic tested target items revealed that movement aids face memory. Participants performed worst in the trials where learning and test modality were inconsistent. The performance difference in the different modality items shows that inclusion of these modalities in the face memory tests may be of benefit for practical purposes and increasing ecological validity. EGEFACE is more similar to everyday face memory tasks than the face memory tests currently used, as it contains both static and dynamic stimuli. It was recently reported that people with extraordinary face memory are being employed in the area of security, in which they are expected to learn and recognize faces from different modalities (Davis et al., 2016, 2018). Therefore, the incorporation of different modalities in EGEFACE provides the opportunity to identify individuals who exhibit superior face memory across various modalities.

Another notable feature of EGEFACE is the utilization of ROC analyses for evaluating performance. This contrasts with currently used face memory tests that typically evaluate performance with percentage accuracy, which has been criticized for its susceptibility to response bias (Masson & Rotello, 2009). Factors such as the characteristics of the stimuli used (e.g., grayscale or animated images, use of faces from multiple races) (Kätsyri, 2018; Slone et al., 2000) and the structure of the test (e.g., repeated exposure to target and distractor faces) (Gold et al., 2007; Kramer & Berry, 2020) might have promoted response bias in participants’ face memory decisions in previous studies (e.g., Bobak et al., 2019; Baudouin et al., 2010). In addition to accounting for these methodological factors, EGEFACE addresses this issue by incorporating a confidence rating scale for responses and by using ROC analysis to assess performance, ensuring that accuracy is measured independently of response bias. The addition of target-absent trials is a crucial aspect of EGEFACE. Previous research has indicated the significance of including target-absent trials in distinguishing between proficient face recognizers and individuals with average abilities (Bobak et al., 2016). Consequently, EGEFACE incorporates both target-present and target-absent trials. Future research is expected to explore the extent to which different trial types contribute to the discrimination of super recognizers.

EGEFACE introduces several valuable additions compared to currently employed face memory tests, but it also contains some limitations. First, all the faces in the test are white-Caucasian face stimuli, which limit the test’s ability to detect super recognizers who can distinguish different race targets/lure faces. Second, even though the internal consistency, split-half reliability, and convergent and divergent validity of the test were examined in this paper, there are other psychometric properties to be examined such as test–retest reliability.^3^ Third, there was a gender imbalance in our samples, with a disproportionately higher number of female participants in both stages of the study. To address this limitation, we compared the test performance of males and females in both test development and replication stages and found no significant differences between the groups in either stage. This suggests that the gender imbalance did not significantly impact the overall findings. Detailed statistical analyses can be found in the supplementary materials. Lastly, one limitation of the current study is the observed difference in performance between participants who completed the test online versus in a lab setting, with lab participants showing slightly higher performance. While this difference was statistically significant, the results of our block difficulty, convergent validity, and item type analyses remained consistent across both online and lab samples. Moreover, this performance difference is not unique to EGEFACE. A similar pattern was observed in the CFMT+, further supporting the reliability of our results. Previous research, such as that by Germine et al. (2012), has also found that online samples can perform either better or worse than lab groups on the CFMT, indicating no systematic difference between online and lab samples in face recognition tests. Furthermore, consistent with our results, Bobak et al. (2023) found that lab participants outperformed online participants across all face recognition and face matching tasks, yet also noted that data collected online were not less reliable or consistent than lab-collected data. Therefore, the observed difference in our study does not appear to affect the overall findings. Detailed statistical analyses can be found in the supplementary materials.

Future research could focus on examining the relationship of EGEFACE with other newly developed face memory tests, self-report measures, and face recognition tasks with high ecological validity by applying it to larger samples. Given preliminary findings that suggest a stronger alignment between EGEFACE and CFMT+ among high performers, assessing the test’s sensitivity with diverse samples, including individuals with developmental prosopagnosia and super recognizers, may provide further insights into its effectiveness across different face recognition abilities. Additionally, analyzing the effect of item type (static/dynamic) during both the learning and test phases could provide valuable insights into capturing and distinguishing the specific face recognition challenges or advantages present in these populations. This multifaceted approach will enhance the understanding of EGEFACE's applicability and reliability in assessing face recognition abilities in diverse contexts.

In conclusion, EGEFACE was developed with the intention of addressing the limitations observed in existing face memory tests. Notable enhancements include the incorporation of static and dynamic stimuli in both learning and test phases, the use of ROC analyses to avoid bias, inclusion of target-absent trials, and the utilization of a face recognition algorithm to modulate test difficulty. Our findings highlight the importance of incorporating diverse modalities in learning and test phases for ecologically valid face memory assessment. Additionally, the successful manipulation of test block difficulty using the face recognition algorithm underscores its efficacy. Future studies on different populations will be effective in assessing and enhancing the reliability and validity of the new test. We hope that EGEFACE will make meaningful contributions to both scientific research and practical applications in the field of face memory.

## Data Availability

The data that support the findings of this study and the supplementary materials are openly available at https://osf.io/gjwk8/ The EGEFACE test is available to the scientific community upon request. Researchers interested in using the test can obtain it by contacting the corresponding author.

## Footnotes

1 There is some variability in memory strength for the target and lure items, and *d'* assumes equal variances for the memory strength distributions for the targets and lures (Rotello et al., 2008). *d'* can only provide bias-free estimates of memory accuracy when the data truly reflect equal variance distributions for the targets and lures (Rotello et al., 2008), which is not the case in most recognition tasks. In fact, memory strength for the target items is assumed to be more variable than that for the lures (i.e., Mickes et al., 2007; Rotello, 2017; Starns & Ratcliff, 2014), resulting in unequal distributions for targets and lures in terms of their levels of variance. On the other hand, AUC provides a nonparametric measure of memory accuracy, without assuming that the distributions of the two classes (targets and lures) have equal variance or any specific shape, thus overcoming possible bias effects and inflated type 1 error rate resulting from the use of d' (Macmillan & Creelman, 2005).

2 When the validity analyses were repeated with the participants who completed all tests, we found consistent results: a moderate positive correlation between EGEFACE and CFMT+ (*r*(96) = .36, *p* < .001) and a significant positive correlation between EGEFACE and KFMT (*r*(96) = .24, *p* < .05).

3 Although we aimed to address the test–retest reliability of EGEFACE in future research, we conducted an initial analysis based on the reviewer's suggestion. Data from 15 participants, with a mean retest interval of 735.27 days, indicated test–retest reliability of *r*(15) = .53, *p* < .05. However, given the variability in retest intervals across participants, we decided not to include this analysis and to pursue a more comprehensive evaluation in future studies.
